# Giant pedunculated oesophageal liposarcomas: A review of literature and resection techniques

**DOI:** 10.1016/j.ijscr.2019.10.006

**Published:** 2019-10-07

**Authors:** Y. Annalisa Ng, June Lee, X.J. Zheng, J.C. Nagaputra, S.H. Tan, S.A. Wong

**Affiliations:** a Department of General Surgery, Changi General Hospital, Singapore; b Department of Pathology, Changi General Hospital, Singapore

**Keywords:** Oesophageal liposarcoma, Giant oesophageal polyp, Resection, Techniques

## Abstract

**Introduction and presentation of case:**

Liposarcomas are rare causes of oesophageal tumours, accounting for <1% of tumours. We present a case of a dedifferentiated oesophageal liposarcoma arising from a giant fibrovascular polyp for which resection was performed via a left cervical oesophagostomy with transgastric retrieval of tumour. We also review the existing literature focusing on discussion of resection techniques.

**Discussion:**

To date, 62 cases of oesophageal liposarcoma have been reported in the literature. They usually occur in males (74.2%), with a median age of 66 years (range 38–84 years). Such tumours present most commonly with dysphagia (69.4%); usually arise from the cervical oesophagus (79%), and are well-differentiated. Treatment options include surgery and recently, endoscopic resection techniques such as submucosal dissection (ESD).

**Conclusion:**

Giant oesophageal liposarcomas are very rare tumours. Such tumours are usually polypoid, arising from a pedicle. As such, resection techniques have shifted away from oesophagectomy to less invasive means such as endoscopic resection or oesophagostomy. Decision on type of resection technique depends on tumour characteristics and location; with the guiding principle being resection with clear margins in order to prevent local recurrence.

## Highlights

• Giant oesophageal liposarcomas, a rare but important cause of oesophageal tumours.
• A review of the current literature focusing on resection techniques.
• Shift in the treatment paradigm in recent years to endoscopic resection techniques.
• Decision on type of resection technique depends on tumour characteristics and location.

## 1 Introduction

Liposarcomas are a very rare cause of tumours in the oesophagus. They account for <1% of oesophageal tumours and usually arise from the upper oesophagus. We present a case of a dedifferentiated liposarcoma arising within a giant fibrovascular polyp and a review focusing on the resection techniques in the literature. This case has been reported in line with the SCARE criteria [18].

## 2 Case

A 54-year-old Chinese gentleman presented to the hospital for palpitations and exertional dyspnoea over 3 months. He was initially reviewed by a cardiologist and a transthoracic echocardiography performed showed a 3.9 cm × 4.2 cm mass posterior to the left atrium. He was also found to be anaemic with a haemoglobin of 5.9 g/dL. Upon further questioning, he denied any symptoms of dysphagia, abdominal or chest discomfort, reflux/regurgitation and respiratory symptoms. He did however note a loss of weight of 5 kg over the same duration with no loss of appetite.

**Fig. 1:**
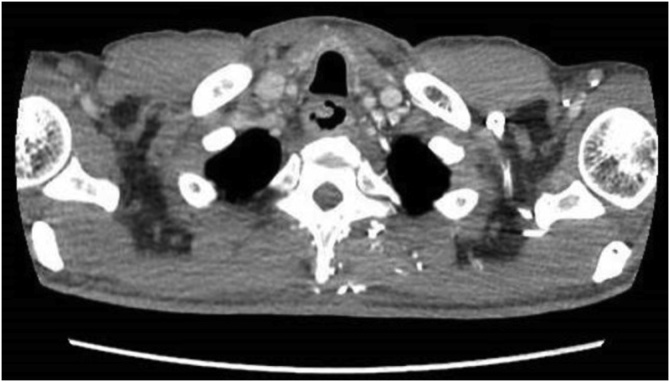
Axial view of CT showing the polyp stalk arising from the right of the cervical oesophagus.

**Fig. 2:**
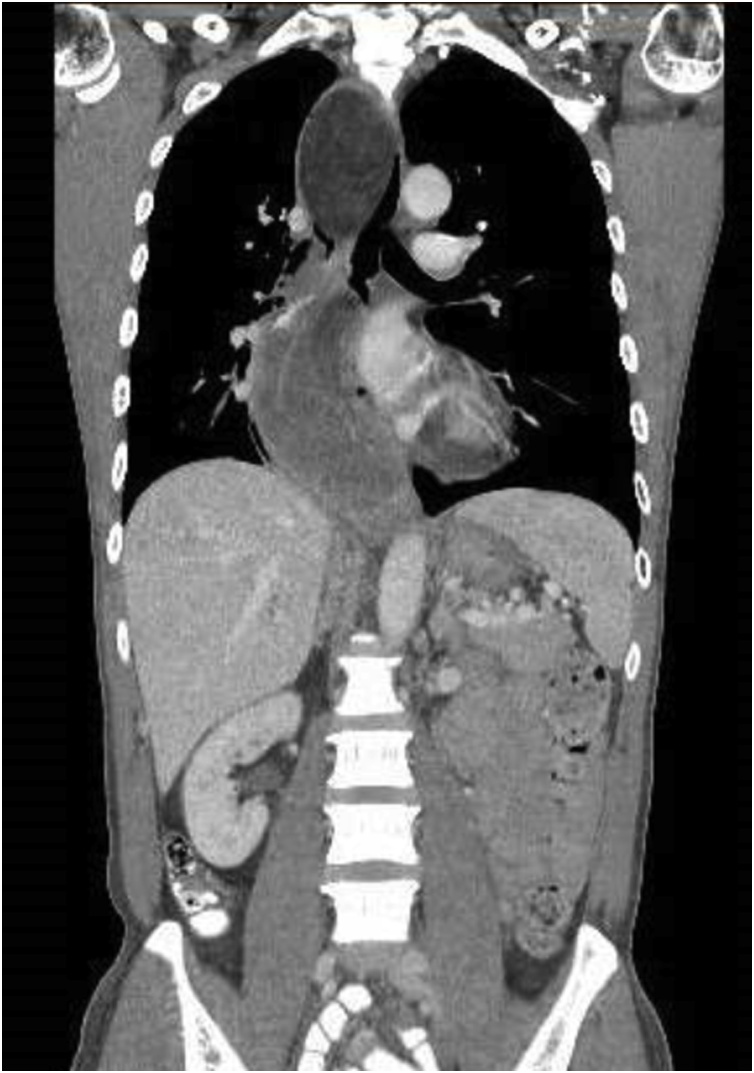
CT showing the heterogenous polypoidal mass.

**Fig. 3:**
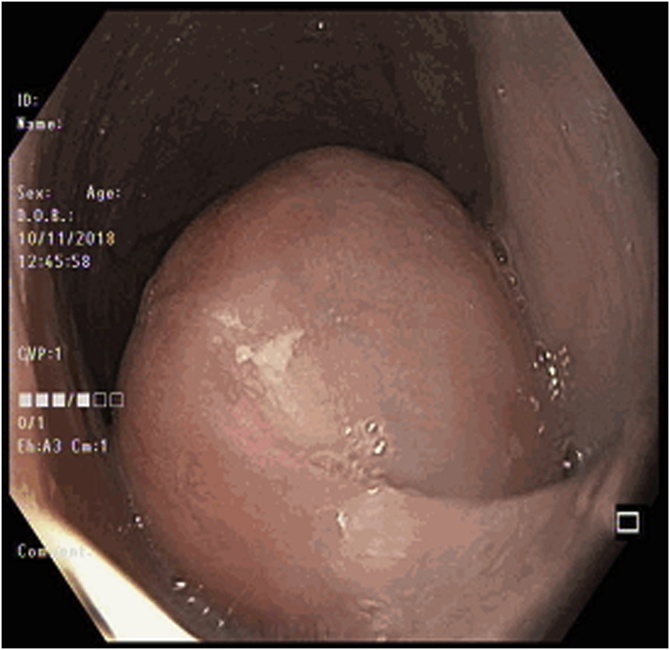
Endoscopic image of the polyp showing its pedicle.

**Fig. 4:**
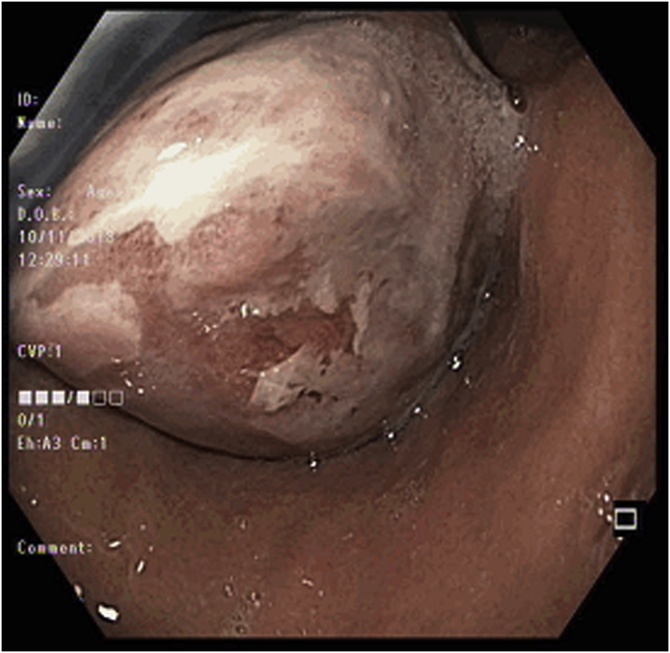
Endoscopic image showing mucosal ulceration at the inferior aspect of the polyp.

A CT scan of the thorax, abdomen and pelvis was performed which showed a markedly dilated oesophagus with large intraluminal masses extending from proximal thoracic oesophagus to cardioesophageal junction (CEJ). There appeared to be 2 masses in close proximity measuring 4.5 cm × 7.2 cm and 5.8 cm × 14.4 cm, with a stalk arising from the cervical oesophagus. The proximal mass was noted to be predominantly fat whilst the distal mass was of a mixed fat and soft tissue attenuation. There was no invasion of adjacent structures and no enlarged lymph nodes or distant metastases. Oesophagogastroduodenoscopy (OGD) performed showed one giant, pedunculated polyp distending the oesophageal diameter and extending from the cervical oesophagus to CEJ. The polyp also appeared to be bilobed with mucosal ulceration at its distal aspect (Fig. 1, Fig. 2, Fig. 3, Fig. 4).

An endoscopic ultrasound (EUS) was also performed, which showed a large submucosal pedunculated multilobed mass with some lobular lipomatous regions (hyperechoic). A vascular stalk was seen at the proximal oesophagus. Core biopsy of the mass however only revealed rare groups of spindle cells with no malignant cells seen.

In view of occult bleeding (and resultant anaemia) from ulceration on the distal polyp, resection was advised. Endoscopic resection was deemed not suitable in view of a highly vascular 1 cm stalk and large size of polyp. He subsequently underwent surgical resection and intraoperative findings were that of a large multiloculated heterogenous oesophageal polyp (arising from a 1 cm-wide stalk in cervical oesophagus) spanning up to the CEJ. Total polyp length was 24 by 6 cm with the cut end of stalk to base of polyp being 3.5 cm.

### 2.1 Surgical technique

**Fig. 5:**
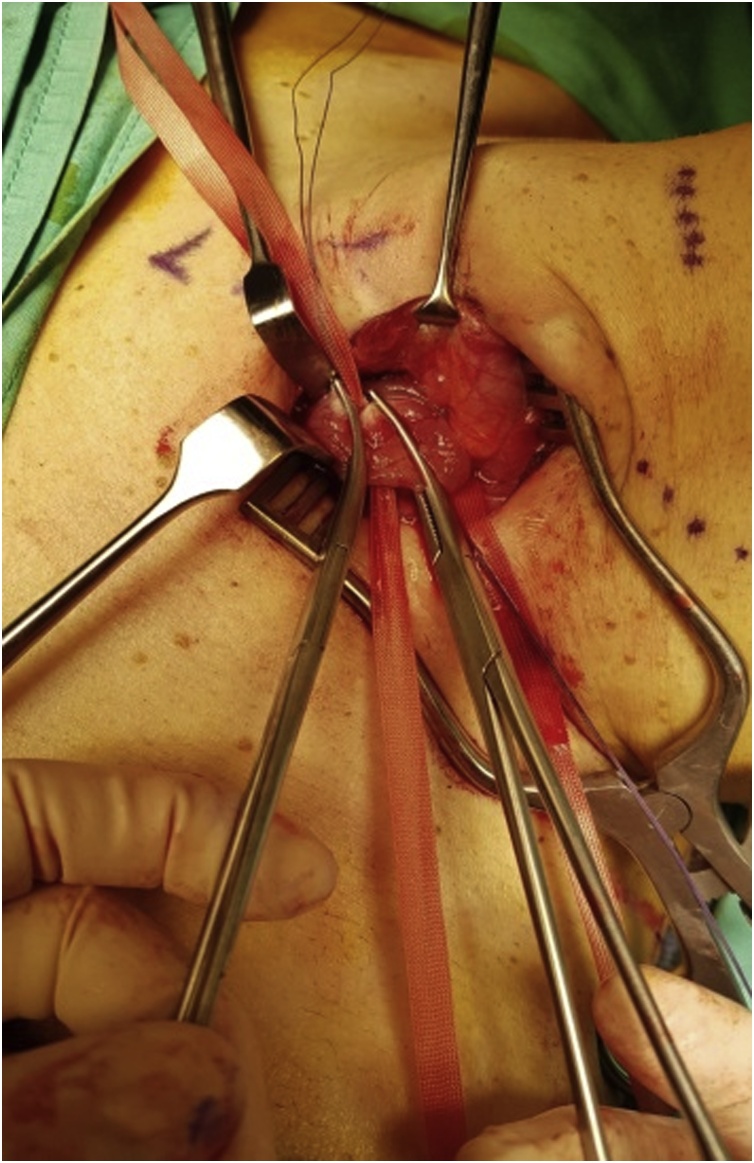
Ligating the polyp stalk between clamps following a left cervical oesophagostomy.

**Fig. 6:**
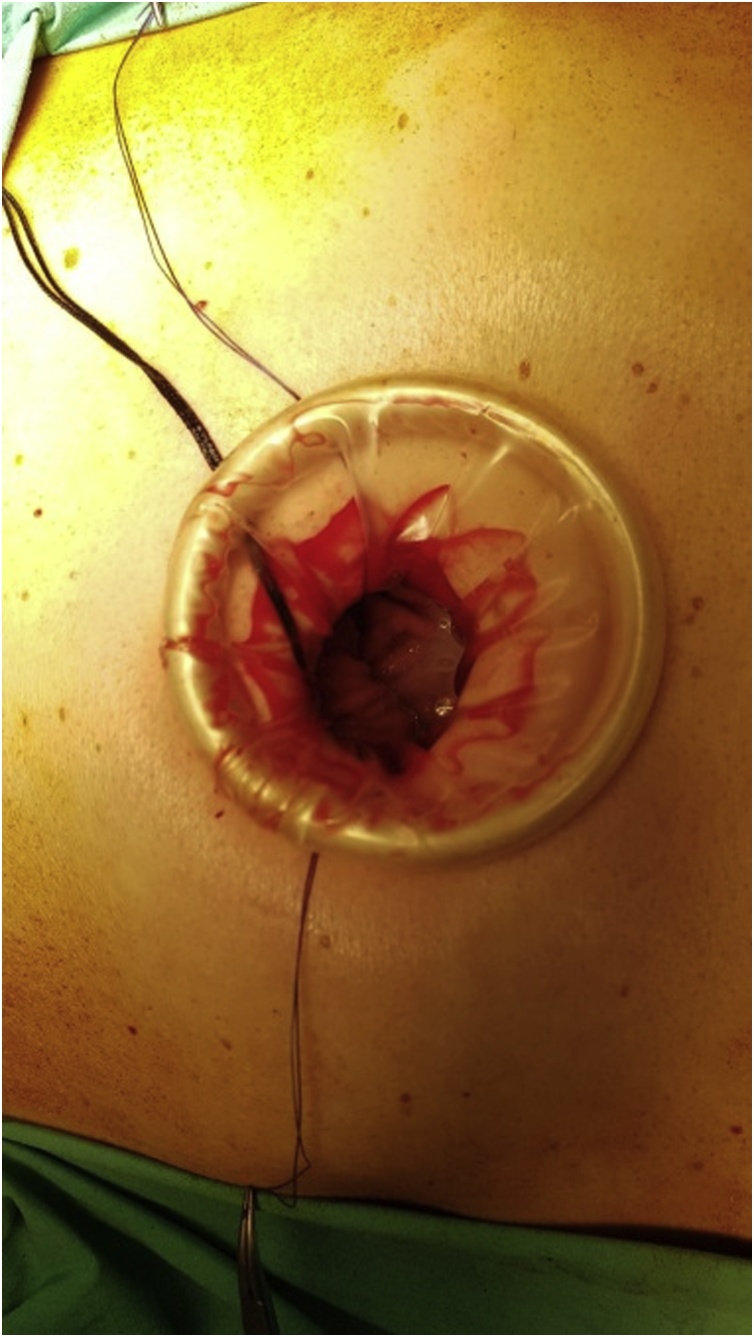
Creation of gastrostomy to retrieve the large polyp.

**Fig. 7:**
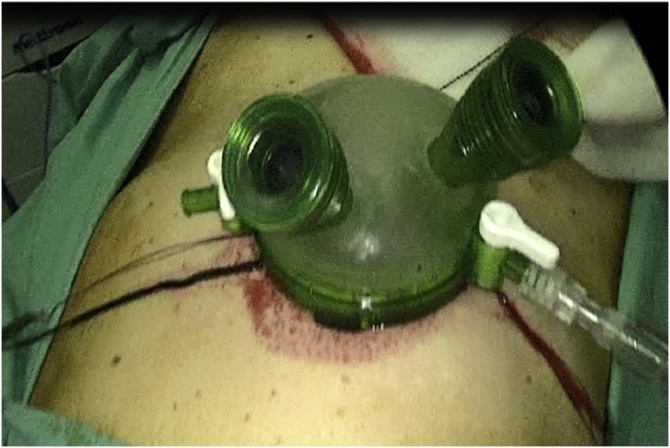
Use of GelPOINT® port to allow transgastric retrieval of polyp.

**Fig. 8:**
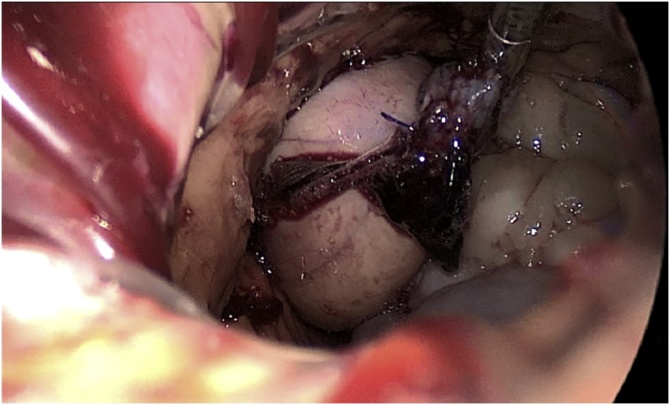
Transgastric retrieval of resected polyp.

**Fig. 9:**
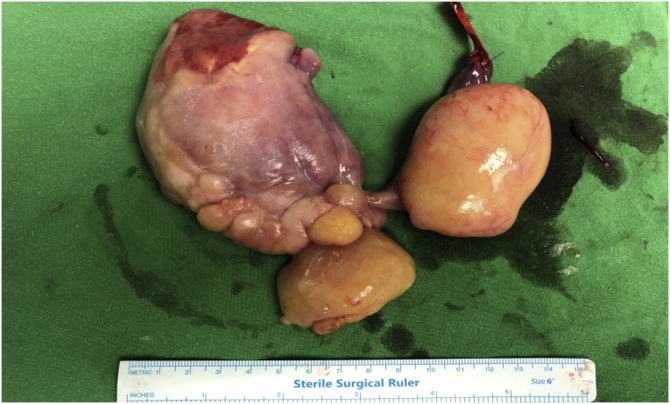
Specimen photo of the giant pedunculated liposarcoma.

The cervical oesophagus was mobilised via a left neck skin crease incision. Longitudinal cervical oesophagostomy was performed on the left side and the polyp stalk on the opposite wall was ligated and oversewn with PDS 3/0 for haemostasis. The polyp diameter was deemed too large for transoral retrieval. A 5 cm left upper quadrant abdominal incision was made and a gastrostomy was performed near the greater curve of the stomach. An Applied medical GelPOINT® port was inserted into the stomach to enable trans-gastric retrieval of the giant oesophageal polyp. Both the cervical oesophagostomy and gastrostomy were closed with PDS 3/0 (Fig. 5, Fig. 6, Fig. 7, Fig. 8, Fig. 9).

The patient’s post-operative recovery was uneventful and he was progressed to diet on POD 3 and discharged well on POD 4. He was well on follow up post-operatively with no evidence of recurrence.

### 2.2 Histology

**Fig. 10:**
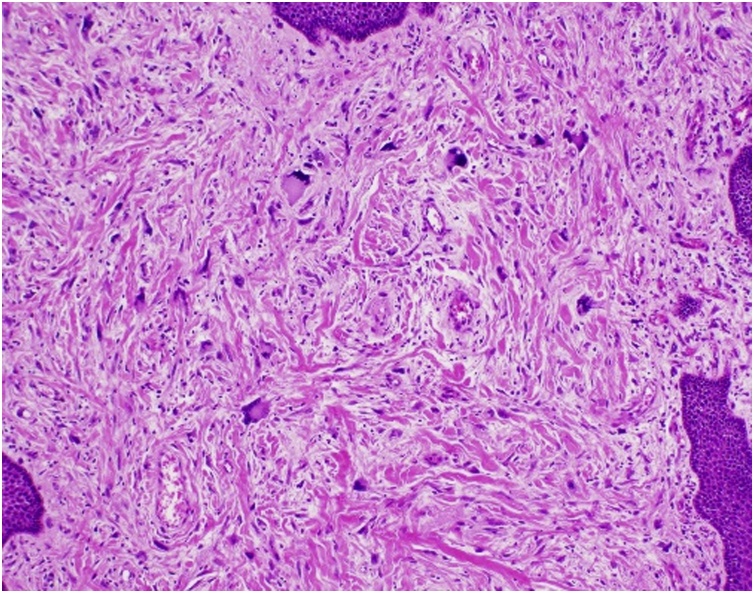
H&E stain (taken at 100x magnification): Squamous-lined mucosa with underlying dedifferentiated adipocyte-poor areas featuring spindle cells with moderate cytological atypia and scattered floret-type giant cells.

**Fig. 11:**
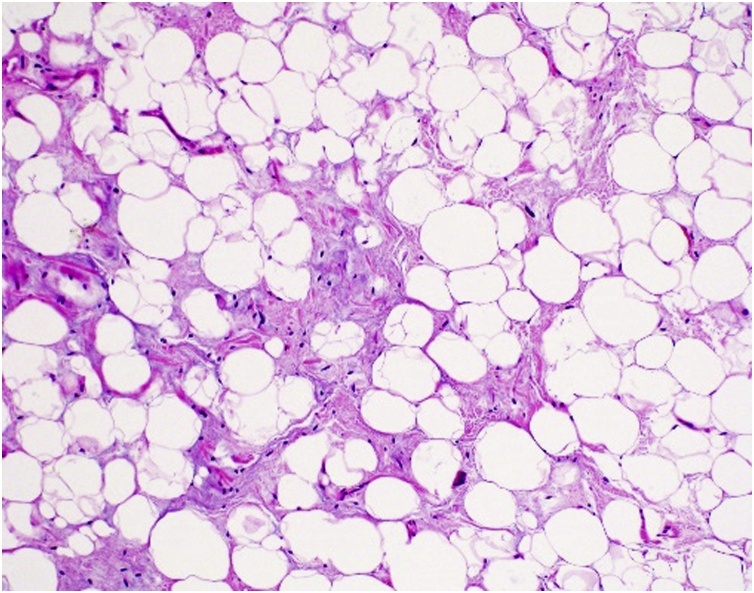
H&E stain (taken at 100x magnification): Mature adipocytes of varying sizes intersected by broad fibrous septa containing scattered atypical cells with enlarged irregular nuclei and hyperchromasia.

Final histology of the oesophageal polyp showed a dedifferentiated liposarcoma arising within a giant fibrovascular polyp, FNCLCC grade 2, measuring 17.5 cm in size. Stalk margin was clear of tumour. FISH (Fluoresence in situ hybridisation) was positive for MDM2. Immunohistochemistry was positive for CD34, SMA and desmin, but negative for STAT 6, ALK-1, S100, AE1/3 and MNF116 (Fig. 10, Fig. 11).

## 3 Literature review

Search in PubMed was performed using the following search string: (oesophagus or esophagus or oesophageal or esophageal liposarcoma) and (Liposarcoma) or (“Esophageal Neoplasms”[MeSH], “Esophageal Diseases”[MeSH] and “Liposarcoma”[MeSH]). All English-language articles and articles from 1983 (first ever case report) to February 2019 were included. The relevant articles found are shown in Table 1. Written informed consent was obtained from the patient for publication of this case report and accompanying images. A copy of the written consent is available for review by the Editor-in-Chief of this journal on request.

**Table 1:** Review of literature.

Author	Age (yrs)/Gender	Presenting symptom	Type of lesion	Tumour Location (Oesophagus)	Histology	Treatment
Aloraini [1]	63/M	Dysphagia	Polypoid	Cervical	Well-differentiated	Endoscopic resection
Arteaga-González	72/M	Dysphagia	Polypoid	Upper	Well-differentiated	Cervical oesophagotomy, Right thoracoscopy
Baca	66/F	Dysphagia	Polypoid	Cervical	Myxoid	Oesophagostomy
Bak	49/F	Dysphagia	Polypoid	Cervical	Well-differentiated	Total Oesophagectomy
Beaudoin	68/F	Dysphagia, vomiting, mass extrusion	Polypoid	Cervical	Well-differentiated	Endoscopic resection
Boggi [2]	50/M	Dysphagia, GI bleed	Polypoid	Cervical	Myxoid	Oesophagostomy, Gastrostomy
Bréhant	70/M	Dysphagia	Polypoid	Upper	Well-differentiated	Cervical oesophagotomy, gastrotomy
Brett	75/M	Dysphagia	Polypoid	Cervical	De-differentiated	Endoscopic (piecemeal)
Chung	56/M	Dysphagia, hoarseness	Polypoid	Cervical	Liposarcoma	Oesophagectomy
Cooper [3]	68/M	Dysphagia	Polypoid	Lower	Myxoid	Subtotal oesophagectomy (invaded muscle layer)
Czekajska-Chehab [4]	56/F	Dyspnoea	Polypoid	Cervical	Well-differentiated	Oesophagectomy
Di Mascio	44/M	Dysphagia, Upper GI bleed	Polypoid	Upper	Well-differentiated	Right thoracotomy and oesophagotomy
Dowli [5]	64/M	Dysphagia	Polypoid	Cervical	Well-differentiated	Cervical oesophagotomy
Garcia	42/M	Dysphagia, vomiting, LOW	Ulcerated, friable mass	Lower	Pleomorphic	Transhiatal Oesophagectomy (tumour bleeding with rupture)
Ginai	53/M	Regurgitation of mass	Polypoid	Cervical	Liposarcoma (not specified)	Oesophagotomy, Carbon dioxide laser (recurrence)
Graham [6]	42 to 84/F(7), M(6)	Not mentioned	All Polypoid	Proximal (7) Middle (1) Distal (1) Non mentioned (4)	Well-differentiated (10) Dedifferentiated (3)	Not mentioned
Hasanabadi	68/M	Dysphagia, Hoarseness	Polypoid (2 lesions)	Cervical	Myxoid	Surgery (not specified)
Jakowski	68/M	Dysphagia	Polypoid	Upper	Rhabdomyomatous, Well-differentiated	Right thoracotomy and oesophagotomy
Liakakos	72/M	Dysphagia, vomiting	Polypoid	Lower	Well-differentiated	Left thoracotomy, oesophagotomy
Lin	51/M	Dysphagia	Submucosal	Upper-middle	Well-differentiated with dedifferentiated component	Mc Keown Oesophagectomy
Mandell	62/F	Dysphagia	Polypoid	Cervical	Well-differentiated	Oesophagotomy
Mansour [7]	53/M	Dyspnoea	Polypoid	Cervical	Myxoid	Oesophagotomy
Maruyama	50/M	Cough	Polypoid	Cervical	Well-differentiated	Oesophagotomy
Masumor	46/F	Protruding tumour in mouth	Polypoid	Cervical	Well-differentiated	Endoscopic resection
McCarthy	61/M	Dysphagia	Polypoid	Cervical	Well-differentiated	Mc Keown Oesophagectomy
Mehdorn	75/M	Dysphagia, LOW	Polypoid	Cervical	Well-differentiated	Thoracotomy, Oesophagectomy
Mica	73/M	Dysphagia, LOW, vomiting	Polypoid	Cervical	Well-differentiated	Oesophagotomy
Moretti	62/F	Dysphagia, regurgitation	Intramural	Thoracic	Liposarcoma (not specified)	Right thoracotomy, oesophagotomy
Myung	67/M	Dysphagia, LOW, vomiting	Polypoid	Cervical	Well-differentiated	Oesophagotomy
Nagahama	68/M	Dysphagia	Polypoid	Cervical	Myxoid	Oesophagotomy
Nakazawa	83/M	Chest discomfort, vomiting	Submucosal	Thoracic	Liposarcoma (not specified)	Subtotal oesophagectomy
Pezzatini	65/M	Dysphagia, LOW	Polypoid	Cervical	Liposarcoma (not specified)	Cervical oesophagotomy and right thoracotomy
Pistorius [8]	66/M	Dysphagia, odynophagia, LOW	Polypoid	Cervical	Well-differentiated	Right thoracotomy and oesophagotomy
Ramacciato	65/M	Dysphagia	Polypoid	Cervical	Liposarcoma (not specified)	Cervical oesophagotomy and right thoracotomy
Riva [9]	81/M	Dysphagia, LOW	Polypoid	Cervical/Hypopharynx	Dedifferentiated	Cervical oesophagotomy
Ruppert-Kohlmayr [10]	72/F	Dysphagia, LOW	Polypoid	Cervical	Well-differentiated	Attempted Endoscopic resection, subsequently surgery (not specified)
Saleh [11]	62/M	Dysphagia, LOW	Polypoid	Cervical	Well-differentiated	Cervical oesophagotomy
Salis	73/M	Dysphagia, vomiting, dyspnoea	Polypoid	Cervical	Well-differentiated	Cervical oesophagotomy
Smith	38/M	Dysphagia, hoarseness, dyspnoea	Polypoid	Cervical	Well-differentiated	McKeown Oesophagectomy
Sui	49/F	Dysphagia	Elliptical mass	Mid-lower	Well-differentiated	Subtotal oesophagectomy
Takiguchi [12]	73/M	Respiratory distress	Polypoid	Cervical	Well-differentiated	Endoscopic resection (ESD), oesophagotomy
Temes	69/M	Dysphagia	Polypoid	Cervical	Well-differentiated	Endoscopic resection (Suture ligation)
Torres-Mora [14]	81/M	Dysphagia, Polyp on screening endoscopy	Polypoid	Cervical	Dedifferentiated	Endoscopic resection
Valiuddin	68/M	Dysphagia, retrosternal pain	Polypoid	Cervical	Rhabdomyomatous (Well-differentiated)	Endoscopic resection (snare and diathermy)
Watkin	50/M	Dysphagia, LOW, Dyspnoea	Submucosal lesion with exophytic component	Lower	Dedifferentiated	Subtotal oesophagectomy
Wil	60/M	Dysphagia	Polypoid	Cervical	Dedifferentiated	Endoscopic resection (diathermy, clips, needle-knife)
Xu	50/M	Dysphagia, vomiting	Submucosal	Upper-middle	Well-differentiated	Oesophagotomy
Yang	49/M	Dysphagia, LOW	Elliptical, submucosal	Upper-middle	Well-differentiated	Right cervical oesophagotomy, thoracotomy and laparotomy
Yates [15]	49/M	Dysphagia, LOW, pain Dysphagia (recurrence)	Polypoid Polypoid	Cervical	Myxoid	Oesophagotomy, thoracotomy (initial op) Mc Keown oesophagectomy (recurrence)
Yo [16]	44/M	Dysphagia	Polypoid	Upper	Well-differentiated	Endoscopic resection (ESD)

## 4 Discussion

Liposarcomas are soft tissue tumours most commonly found in the retroperitoneum. It is rarely found in the gastrointestinal tract, especially the oesophagus, and it is estimated to account for less than 1% of oesophageal neoplasms. Ever since the first report by Mansour [7] in 1983, there has been a total of 62 patients reported to date, with the largest series of 13 patients coming from Graham et al. [6]

A literature review by Dowli et al. [5] found that there were 5 types of oesophageal liposarcomas described (well differentiated, myxoid, round cell, pleomorphic, dedifferentiated), of which the majority were well-differentiated. Dedifferentiated liposarcomas account for an even smaller proportion of oesophageal sarcomas, are usually high grade, have a propensity for local recurrence [13,14] and may metastasize, although recurrence is less common in sarcomas arising from non-retroperitoneal locations. Graham et al. [6] reviewed their own series of 13 patients previously described as giant fibrovascular polyps of the oesophagus, and found that all cases showed MDM2 amplification, confirming diagnoses of well-differentiated (majority) and dedifferentiated liposarcomas.

Review of the literature also revealed that the majority of oesophageal liposarcomas are polypoid with the stalk arising most commonly from the cervical oesophagus. These tumours have the potential to grow to large sizes resulting in obstruction. Reported tumour sizes ranged from 4 cm to a maximum length of 33 cm [8], with the majority being more than 10 cm in length. The most common presentation is dysphagia, and other less common presentations include regurgitation of polyp, bleeding/anaemia from tumour ulceration, foreign body sensation, airway obstruction and even asphyxiation [2,3,4,7,15].

The mainstay of treatment is resection with clear margins. Open surgery has classically been the standard of treatment, but over the past decade with advances in technology, endoscopic resection has become a viable option for tumour resection. Endoscopic resection confers the benefit of being less morbid and invasive compared with conventional surgery, especially since the majority of such tumours are pedunculated giant oesophageal polyps arising from a single stalk. Endoscopic techniques described in the literature include (a) using a stabilising retraction suture placed with an endoscopic suturing device followed by division of stalk using ultrasonic shears [1], (b) using a snare with cutting and coagulation [10,13] (c) endoscopic submucosal dissection (ESD) with knife [12,16], and (d) application of hemoclips following diathermy [9]. The subsequent tumour then can either be removed transorally or retrieved via an oesophagotomy or laparotomy if it is too large. Decision for endoscopic resection depends on a few factors: (a) whether the giant polyp is pedunculated or sessile, (b) whether there is a single stalk or multiple stalks, and (c) whether there are large feeding vessels within the stalk. In this case, endoscopic resection of the polyp was not advised in view of EUS findings of a highly vascular polyp stalk with higher risk of bleeding, and the large size of the multilobulated polyp which would preclude the use of a snare.

In cases where endoscopic resection is not an option, surgery would be the definitive mode of resection. Oesophagostomy, oesophagectomy and laparotomy for resection and retrieval of the tumour have been described in the literature. A systematic review by Dowli et al. [5] of 40 cases of oesophageal liposarcomas showed that oesophagostomy was the main type of surgical resection (41.9%), followed by oesophagectomy (25.8%) and thoracotomy (16.1%). Main reason for oesophagectomy was the presence of a submucosal (rather than polypoid, pedunculated) large tumour, with a need for clear resection margins to reduce the risk of local recurrence. For resection via oesophagostomy in those with pedunculated tumours, the oesophagostomy needs to be carefully placed away from the side of the polyp stalk to enable adequate control prior to resection. Resection of the stalk can be performed via stapling devices eg. EndoGIA [8,11], or via simple transfixion and ligation with sutures. In our case, a cervical oesophagostomy was performed to ligate the polyp stalk, followed by a small transverse incision in the left upper abdomen for transgastric retrieval of the tumour in view of size and to avoid tumour rupture and seeding of tumour cells.

Following complete resection with clear margins, most authors advocate surveillance with OGD and/or imaging such as computed tomography (CT) scans as such tumours have a propensity for local recurrence. The role of adjuvant radiotherapy and is still controversial and can be complicated by side effects such as constrictive pericarditis, radiation pneumonitis and lung fibrosis [17].

## 5 Conclusion

Giant oesophageal liposarcomas are very rare tumours. The majority present with dysphagia and such tumours are usually polypoid and arise from a pedicle at the cervical oesophagus. As such, resection techniques have shifted from oesophagectomy to less invasive means such as endoscopic resection or oesophagostomy. Decision on type of resection technique depends on tumour characteristics and location; with the guiding principle being resection with clear margins in order to prevent local recurrence.

## Funding

No sources of funding.

## Ethical approval

This study is exempt from ethical approval as we have obtained informed consent from the patient and this is a report of the patient’s management previously and does not change his existing management.

## Consent

Informed consent has been obtained from this patient.

## Registration of research studies

This is not needed as per http://www.researchregistry.com (“We do not register case reports that are not first-in-man or animal studies”).

## Guarantor

Ng YA.

Lee J.

## Provenance and peer review

Not commissioned, externally peer-reviewed.

## CRediT authorship contribution statement

**Y. Annalisa Ng:** Conceptualization, Visualization, Writing - original draft, Writing - review & editing. **June Lee:** Writing - review & editing, Supervision. **X.J. Zheng:** Writing - review & editing. **J.C. Nagaputra:** Resources. **S.H. Tan:** Resources. **S.A. Wong:** Supervision.

## Declaration of Competing Interest

There are no conflicts of interest for this case report.
